# MetaMDA: explainable prediction of microbe–drug association utilizing random walks on a microbe–metabolite–drug heterogeneous network

**DOI:** 10.1093/bioinformatics/btaf649

**Published:** 2025-12-01

**Authors:** Qi Wang, Shuting Chen, Xintian Miao, Yuntao Liu, Bingqiang Liu

**Affiliations:** School of Mathematics, Shandong University, Jinan, Shandong 250100, China; School of Mathematics, Shandong University, Jinan, Shandong 250100, China; School of Mathematics, Shandong University, Jinan, Shandong 250100, China; School of Mathematics, Shandong University, Jinan, Shandong 250100, China; School of Mathematics, Shandong University, Jinan, Shandong 250100, China; Shandong National Center for Applied Mathematics, Jinan, Shandong 250100, China; State Key Laboratory of Cryptography and Digital Economy Security, Shandong University, Jinan, Shandong 250100, China

## Abstract

**Motivation:**

Human-associated microbes play a critical role in physiological processes and disease development, including cancer. Predicting microbe–drug associations (MDAs) can aid drug discovery and personalized medicine. However, existing methods cannot predict MDAs involving microbes or drugs absent from labeled data, and they fail to model the underlying biological mechanisms between microbes and drugs. To address these limitations, we propose a novel computational framework, named MetaMDA, for predicting MDAs by performing random walks on a microbe–metabolite–drug heterogeneous network. MetaMDA first constructs a heterogeneous graph that integrates microbes, metabolites, and drugs, enabling the modeling of complex biological interactions. A random walk algorithm with tailored transition probabilities is subsequently applied to the graph, effectively capturing features from multiple node types on a unified scale.

**Results:**

Experimental results across multiple datasets demonstrate that MetaMDA consistently outperforms state-of-the-art methods, achieving an average improvement of 26%. Notably, we show MetaMDA’s unique ability to predict MDAs involving microbes or drugs absent from labeled data, as illustrated by associations related to acarbose. Furthermore, mechanistic analysis of MetaMDA provides biological explanations for the associations between *Escherichia coli* and escitalopram, highlighting its potential to reveal a deeper mechanistic understanding of microbe–drug associations.

**Availability and implementation:**

The code and datasets are available on Zenodo https://doi.org/10.5281/zenodo.17348446 and GitHub https://github.com/wqlyt17/MetaMDA.

## 1 Introduction

The microorganisms that reside in the human body are involved in various crucial physiological processes and contribute to the development, diagnosis, and treatment of human diseases (Wang *et al.* 2017, Sepich-Poore *et al.* 2021, Wang *et al.* 2023). Importantly, extensive studies have demonstrated the intricate interactions between microbes and drugs, which are essential to comprehending the heterogeneity in drug responses among individuals (Zhao *et al.* 2023). Microbes play a significant role in modulating the absorption, distribution, metabolism, and excretion of drugs (Haiser *et al.* 2014, Enright *et al.* 2016, Violi *et al.* 2016, Zhao *et al.* 2023). Conversely, drugs can also modify the composition and function of microbial communities, leading to changes in microbial metabolism and immune response (Forslund *et al.* 2015, Zhao *et al.* 2023). Therefore, identifying the relationship between microbes and drugs is important for further explaining the individual variability in drug response and enhancing patient outcomes. Given the experimental difficulties in the standard collection and sequencing of clinical samples (Zhao *et al.* 2023), increasing efforts have been devoted to computationally predicting the microbe–drug associations (MDAs), which are essential for investigating the underlying mechanisms of microbe–drug interactions. The microbe–drug association prediction problem can be formulated as a link prediction task, aiming to identify previously unobserved MDAs within the microbe–drug network, which is constructed based on labeled MDAs from MDAD (Sun *et al.* 2018), aBiofilm (Rajput *et al.* 2018), and MASI (Zeng *et al.* 2021, Wang *et al.* 2025) datasets.

Several studies have been proposed to identify the potential microbe–drug associations. For example, HMDAKATZ constructs a heterogeneous microbe–drug network using similarity networks and known associations and predicts MDAs based on the KATZ measure (Zhu *et al.* 2019). GCNMDA develops a heterogeneous network for drugs and microbes and utilizes a GCN-based framework enhanced with a conditional random field and an attention mechanism to identify MDAs (Long *et al.* 2020a). EGATMDA introduces an ensemble framework of graph attention networks, integrating hierarchical attention mechanisms and meta-path-based virtual associations to predict MDAs (Long *et al.* 2020b). Deng *et al.* proposed Graph2MDA, which constructs multimodal attribute graphs as inputs for variogram autoencoders, enabling detailed discovery of individual nodes and the overall graph structure (Deng *et al.* 2022). SCSMDA is an MDA prediction method that integrates structure-enhanced contrastive learning and self-paced negative sampling to learn discriminative embeddings and improve prediction accuracy (Tian *et al.* 2023b). GARFMDA integrates graph attention networks with a bilayer random forest to predict potential MDAs by extracting and filtering key feature representations (Kuang *et al.* 2024).

However, despite advances in computational methods for predicting MDAs, several limitations remain. On the one hand, existing approaches struggle to predict MDAs involving microbes or drugs that are absent from labeled MDA datasets, significantly limiting the identification of more comprehensive MDAs. On the other hand, current methods primarily focus on solving the computational link prediction problem formulated by MDA prediction, often neglecting the underlying biological interaction mechanisms between microbes and drugs. Fortunately, many studies have demonstrated that metabolites can act as mediators in complex drug-microbe interactions (Zimmermann *et al.* 2021, Lindell *et al.* 2022, Bai *et al.* 2023, Zhao *et al.* 2023). For example, the microbiome-produced metabolite p-cresol has been shown to interfere with acetaminophen clearance (Zimmermann *et al.* 2021). Additionally, non-steroidal anti-inflammatory drugs (NSAIDs) can disrupt the gut microbiota metabolism of bile acids (BAs), leading to alterations in the gut microbial community (Bai *et al.* 2023). Therefore, by incorporating a metabolite space associated with drugs and microbes, we can introduce new drugs and microbes not previously involved in labeled MDAs, thereby enhancing the biological interpretability and comprehensiveness of identified MDAs.

In this study, we proposed an explainable computational framework utilizing random walks on a microbe–metabolite–drug heterogeneous network to predict MDAs. By incorporating a metabolite space associated with drugs and microbes, we first constructed a heterogeneous graph to represent the relationships among microbes, metabolites, and drugs. This network captures metabolic activity associations between microbes, metabolites, and drugs, as well as enzymatic reaction relationships among metabolites. Based on this heterogeneous network, we designed a novel random walk algorithm to effectively capture the features of different node types on a uniform scale. The algorithm defines transition probabilities between various node pairs, enabling an explainable and biologically meaningful walk. Subsequently, a node-type-aware heterogeneous Skip-gram approach was used to learn embedding representations from the random walk-generated node sequences. Finally, these embeddings were used to train an XGBoost classifier for accurate MDA prediction. As a result, our method consistently outperforms all state-of-the-art approaches across multiple datasets, achieving an average relative improvement of 26%, which demonstrates the robustness and effectiveness of MetaMDA. We conducted ablation studies and parameter sensitivity, which validated the contributions of MetaMDA’s key components. More importantly, we demonstrated MetaMDA’s unique capability to predict MDAs involving microbes or drugs that were absent from the labeled MDAs, as exemplified by the case study on acarbose. Furthermore, analysis of the random walk node sequences containing *Escherichia coli* revealed that the synergistic effect of *E*. *coli*-derived glutamate and escitalopram influences neurotransmitter balance, providing a biological explanation for the association between *E*. *coli* and escitalopram. Unlike existing methods that primarily treat MDA prediction as a computational link prediction task, our approach offers deeper insights into the underlying biological interaction mechanisms between microbes and drugs.

## 2 Materials and methods

**Figure 1.**
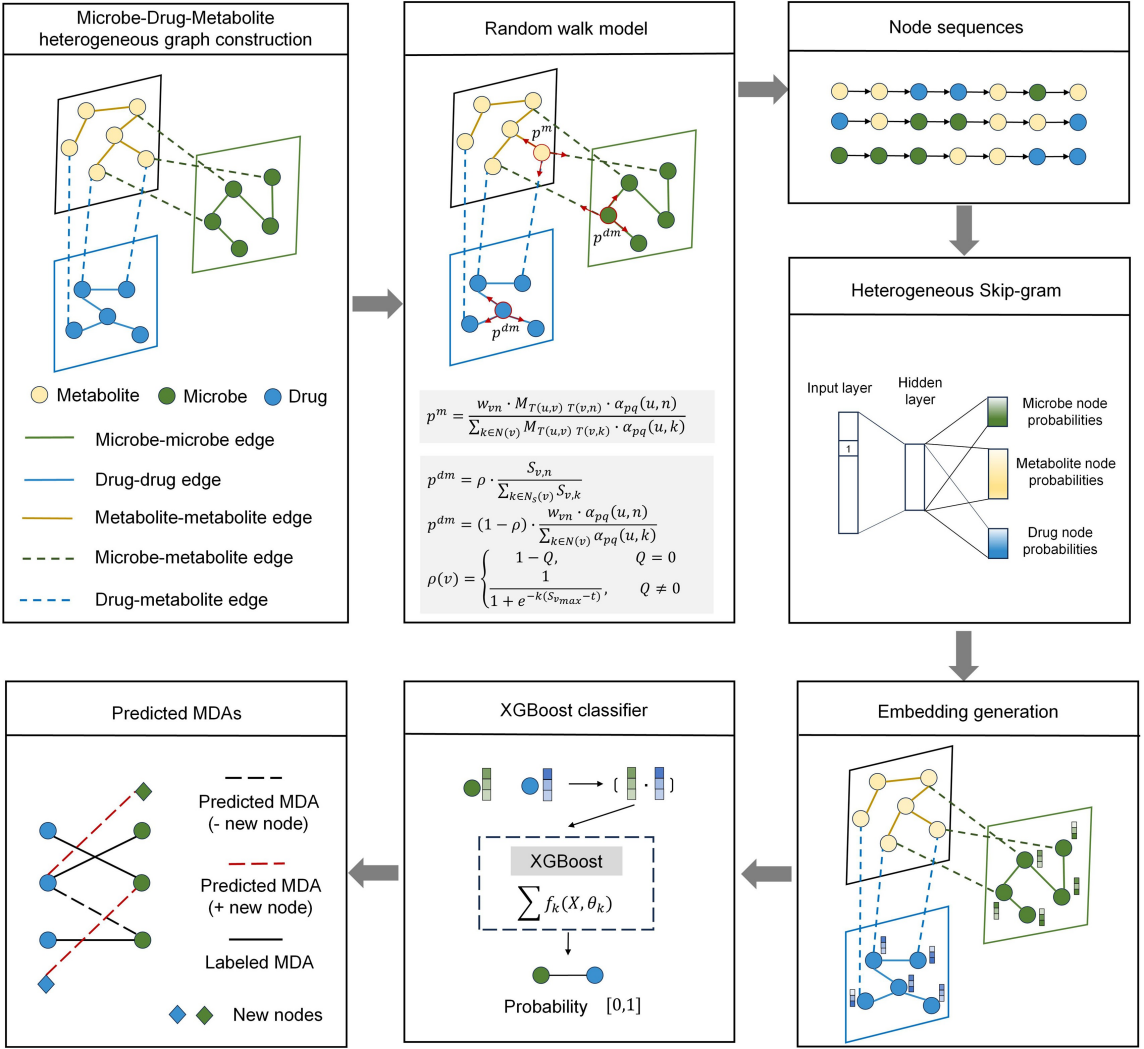
The framework of MetaMDA. MetaMDA first constructs a heterogeneous graph to represent the relationships among microbes, metabolites, and drugs. Based on this heterogeneous network, a novel random walk algorithm is designed to effectively capture the features of different node types on a unified scale. For each node in the heterogeneous network, 100 walks of length 10 are generated. Subsequently, a node-type-aware heterogeneous Skip-gram model is used to learn embedding representations from the node sequences generated by random walks. Finally, these embeddings are used to train an XGBoost classifier for accurate MDA prediction. MetaMDA demonstrates a unique ability to predict MDAs involving microbes or drugs that are absent from the labeled MDA dataset. “+ new node” refers to MDAs involving microbes or drugs not present in the labeled dataset, while “– new node” refers to MDAs involving microbes or drugs that are already included in the labeled dataset.

MetaMDA is a novel computational framework for predicting MDAs by utilizing random walks on a microbe–metabolite–drug heterogeneous network (Fig. 1). This framework uses a random walk model to capture the features of different node types within the heterogeneous graph and utilizes an XGBoost classifier to predict MDAs. MetaMDA mainly consists of data collection, microbe–metabolite–drug heterogeneous graph construction, random walk modeling, embedding generation, and MDA prediction. In the following sections, we will introduce each of these steps in detail.

### 2.1 Data collection

Three public experimentally confirmed microbe–drug association datasets, which are Microbe–Drug Association Database (MDAD) (Sun *et al.* 2018), aBiofilm (Rajput *et al.* 2018), and MASI (Zeng *et al.* 2021, Wang *et al.* 2025), are used for training and evaluating the MDA prediction performances of the proposed and baseline models. MDAD is a collection of clinically or experimentally supported associations between microbes and drugs, collecting 2470 entries that include 1373 drugs and 173 microbes from multiple drug databases and related publications. aBiofilm is a comprehensive database offering biological, chemical, and structural information on 1720 anti-biofilm agents targeting over 140 microbial species, encompassing 2884 microbe–drug associations. MASI contains 8760 microbe–drug associations involving 707 drugs and 525 microorganisms.

### 2.2 Microbe–metabolite–drug heterogeneous graph construction

A microbe–metabolite–drug (MMD) heterogeneous graph G=(V,E,P,Q) was constructed, where V is the node-set, E is the edge set, and P and Q represent node type and edge type respectively. The heterogeneous graph G was composed of three types of node (microbe nodes, drug nodes, and metabolite nodes) and five types of edge (microbe–microbe edges, drug–drug edges, metabolite–metabolite edges, microbe–metabolite edges, and drug–metabolite edges). Mathematically, $V=V^{s}\cup V^{m}\cup V^{d}$, where $V^{s}=\{v_{i}^{s}|i=1,2,\ldots ,N_{s}\}$ denotes all microbe nodes, $V^{m}=\{v_{j}^{m}|j=1,2,\ldots ,N_{m}\}$ denotes all metabolite nodes, $V^{d}=\{v_{k}^{d}|k=1,2,\ldots ,N_{d}\}$ denotes all drug nodes. $E=E^{ss}\cup E^{sm}\cup E^{mm}\cup E^{dm}\cup E^{dd}$. $E^{ss}=\{e_{ij}^{ss}|i,j\in \{1,2,\ldots ,N_{s}\},\;\mathrm{and}\;{SS}_{i,j}>0\}\;$denotes the set of microbe–microbe edges, where $e_{ij}^{ss}$ represents the edge between microbe nodes $v_{i}^{s}\in V^{s}$ and $v_{j}^{s}\in V^{s}$, and SS is the microbe–microbe matrix. $E^{sm}=\{e_{ij}^{sm}|i\in \{1,2,\ldots ,N_{s}\},\;j\in \{1,2,\ldots ,N_{m}\}\;\mathrm{and}\;{SM}_{i,j}=1\}$ denotes the set of microbe–metabolite edges, where $e_{ij}^{sm}$ represents the edge between microbe $v_{i}^{s}\in V^{s}$ and metabolite $v_{j}^{m}\in V^{m}$, and SM is the microbe–metabolite matrix. $E^{mm}=\{e_{ij}^{mm}|i,j\in \{1,2,\ldots ,N_{m}\}\;\mathrm{and}\;{MM}_{i,j}=1\}$ denotes the set of metabolite–metabolite edges, where $e_{ij}^{mm}$ represents the edge between metabolite nodes $v_{i}^{m}\in V^{m}$ and $v_{j}^{m}\in V^{m}$, and MM is the metabolite–metabolite matrix. $E^{dm}=\{e_{ij}^{dm}|i\in \{1,2,\ldots ,N_{d}\},\;j\in \{1,2,\ldots ,N_{m}\}\;\mathrm{and}\;{DM}_{i,j}=1\}$ denotes the set of drug–metabolite edges, where $e_{ij}^{dm}$ represents the edge between drug node $v_{i}^{d}\in V^{d}$ and metabolite node $v_{j}^{m}\in V^{m}$, and DM is the drug–metabolite matrix. $E^{dd}=\{e_{ij}^{dd}|i,j\in \{1,2,\ldots ,N_{d}\},\;\mathrm{and}\;{DD}_{i,j}>0\}\;$denotes the set of drug–drug edges, where $e_{ij}^{dd}$ represents the edge between drug nodes $v_{i}^{d}\in V^{d}$ and $v_{j}^{d}\in V^{d}$, and DD is the drug–drug matrix. The details of the constructed MMD heterogeneous graph are provided in Table S1, available as supplementary data at *Bioinformatics* online.

#### 2.2.1 Metabolite–metabolite edges

The edges between two metabolites are based on the metabolite-to-metabolite-based enzymatic reactions from the KEGG database (Shen *et al.* 2019). If two metabolites are reaction-paired, an edge is formed between them. Specifically, when the substrate and product metabolites of a metabolic reaction are structurally similar, they are defined as reaction-paired metabolites (Shen *et al.* 2019). We denoted the metabolite–metabolite matrix is MM. ${MM}_{i,j}=1$ indicates that there is an edge between metabolite $v_{i}^{m}$ and metabolite $v_{j}^{m}$, while ${MM}_{i,j}=0$ indicates that there is no edge between them.

#### 2.2.2 Microbe–metabolite edges

The edges between microbes and metabolites are based on their production and consumption relationships. If a microbe produces or consumes a metabolite, an edge is formed between them. In this study, we use the public NJS16 database (Sung *et al.* 2017) to evaluate the production and consumption relationships of a pair of microbe and metabolite. The NJS16 database collects experimentally verified metabolic interactions from the literature, listing the compounds produced or consumed by each microbial species. We denoted the microbe–metabolite matrix as SM, where each row represents a microbe, and each column represents a metabolite. ${SM}_{i,j}=1$ indicates that there is an edge between microbe $v_{i}^{s}$ and metabolite $v_{j}^{m}$, while ${SM}_{i,j}=0$ indicates that there is no edge between them.

#### 2.2.3 Drug–metabolite edges

The edges between metabolites and drugs are derived from the metabolite-drug associations from the work of Xiuhong *et al.* (Li *et al.* 2023) based on the Human Metabolome Database (HMDB) (Wishart *et al.* 2022) and DrugBank (Wishart *et al.* 2018). If a metabolite’s concentration changes following drug intake, it is considered that the metabolite has a relationship with the drug, which forms an edge between them. Specifically, HMDB provides comprehensive reference information about the human metabolome. DrugBank is the most comprehensive web resource about drugs. We denoted the drug–metabolite matrix as DM, where each row represents a drug, and each column represents a metabolite. ${DM}_{i,j}=1$ indicates that there is an edge between drug $v_{i}^{d}$ and metabolite $v_{j}^{m}$, while ${DM}_{i,j}=0$ indicates that there is no edge between them.

#### 2.2.4 Microbe–microbe edges

The edges between two microbes are based on the average similarity of microbes from two aspects, including Gaussian interaction profile kernel-based similarity and functional similarity. We measure all the similarities of all microbe pairs. If the average similarity between two microbes $v_{i}^{s}$ and $v_{j}^{s}$ is over similarity threshold α, then a microbe–microbe edge between them is generated, denoted as ${SS}_{i,j}=\mathrm{similarity}$.

The 1st similarity between microbes is the Gaussian interaction profile kernel-based similarity. The basic assumption for this type of similarity is that similar microbes interacting with similar drugs or metabolites will have similar profiles. This similarity measure includes the Gaussian interaction profile kernel-based similarity derived from the microbe–drug matrix as well as that from the microbe–metabolite matrix. Specifically, suppose the microbe–drug association matrix is I, where each row represents a microbe, each column represents a drug, and the entry represents whether the microbe interacts with the drug. Here, we denote the ith and jth row in the microbe–drug association matrix I as $I(v_{i}^{s})$ and $I(v_{j}^{s})$. Thus, the Gaussian interaction profile kernel-based similarity for microbe $v_{i}^{s}$ and $v_{j}^{s}$ is calculated as:


$$GM(v_{i}^{s},v_{j}^{s})=\exp(-\eta_{s}{\left||I(v_{i}^{s})-I(v_{j}^{s})|\right|}^{2})$$

where $\eta_{s}=\eta_{s}^{\prime }/(\frac{1}{N_{s}}\sum_{i=1}^{N_{s}}{\left||I(v_{i}^{s})|\right|}^{2})$ is the normalized kernel bandwidth, $\eta_{s}^{\prime }$ is always set to 1; $N_{s}$ is the number of rows in matrix I. For the microbe–metabolite matrix SM, the calculation of the Gaussian interaction profile kernel-based similarity is similar (Supplementary Method S1, available as supplementary data at *Bioinformatics* online).

The 2nd similarity between microbes is based on functional similarity, which includes both genomic and evolutionary similarities. To quantify genomic similarity, we utilized $1-d_{\mathrm{mash}}(v_{i}^{s},\;v_{j}^{s}),\;$where $d_{\mathrm{mash}}(v_{i}^{s},\;v_{j}^{s})\;$represents the mash score between microbes $v_{i}^{s}$ and $v_{j}^{s}$, a measure used to profile genome distance (Ondov *et al.* 2016). The mash score is calculated using the Mash tool, which includes a pairwise mutation distance and *P* value significance test, enabling the efficient clustering and search of massive sequence collections (Ondov *et al.* 2016). Additionally, we calculated the evolutionary similarity using $\frac{1-d_{p}(v_{i}^{s},\;v_{j}^{s})}{\max_{d_{p}}}$, where $d_{p}(v_{i}^{s},\;v_{j}^{s})\;$represents the patristic distance between microbes $v_{i}^{s}$ and $v_{j}^{s}$. The patristic distance between microbes $v_{i}^{s}$ and $v_{j}^{s}$ is the sum of the lengths of the branches that link $v_{i}^{s}$ and $v_{j}^{s}$ in a phylogenetic tree, which is used to profile evolutionary differences. We calculated the patristic distance by utilizing the Biopython module (Cock *et al.* 2009).

#### 2.2.5 Drug–drug edges

The edges between two drugs are based on the average similarity of drugs from two aspects, including Gaussian interaction profile kernel-based similarity and functional similarity. We measure all the similarities of all drug pairs. If the average similarity between two drugs $v_{i}^{d}$ and $v_{j}^{d}$ is over similarity threshold α, then a drug–drug edge between them is generated, denoted as ${DD}_{i,j}=\mathrm{similarity}$.

The 1st similarity between drugs is the Gaussian interaction profile kernel-based similarity, which is computed in the same way as the Gaussian interaction profile kernel-based similarity of microbes. This similarity measure encompasses the Gaussian interaction profile kernel-based similarity derived from the microbe–drug matrix as well as that from the microbe–metabolite matrix. Specifically, we denote the ith and jth column in the microbe–drug association matrix I as $I(v_{i}^{d})$ and $I(v_{j}^{d})$. Thus, the Gaussian interaction profile kernel-based similarity for drug $v_{i}^{d}$ and $v_{j}^{d}$ is calculated as:


$$GM(v_{i}^{d},v_{j}^{d})=\exp(-\eta_{d}{\left||I(v_{i}^{d})-I(v_{j}^{d})|\right|}^{2})$$

where $\eta_{d}=\eta_{d}^{\prime }/(\frac{1}{N_{d}}\sum_{i=1}^{N_{d}}{\left||I(v_{i}^{d})|\right|}^{2})$ is the normalized kernel bandwidth, $\eta_{d}^{\prime }$ is always set to 1; $N_{d}$ is the number of columns in matrix I. For the drug–metabolite matrix DM, the calculation of the Gaussian interaction profile kernel-based similarity is similar (Supplementary Method S2, available as supplementary data at *Bioinformatics* online).

The 2nd similarity between drugs is based on functional similarity. To assess functional similarity between drugs, we utilized RDKFingerprints. Notably, RDKFingerprints primarily capture the topological structure of drug molecules, effectively encoding atomic connectivity, substructure patterns, and topological path features (Landrum 2013). Drugs are first represented in the canonical Simplified Molecular Input Line Entry System (SMILES) format (Weininger 1988) and then converted into RDKFingerprints using RDKit (Landrum 2013). The Tanimoto coefficient, appropriate for fingerprint-based similarity calculations, was subsequently computed for each pair of drugs based on their RDKFingerprints. We calculated the functional similarity of drugs by utilizing the RDKit library in Python.

### 2.3 Random walk modeling

After constructing the MMD heterogeneous graph, we designed a novel random walk model to effectively capture the relational information among microbes, metabolites, and drugs within the graph. The model generates explainable and biologically meaningful walk sequences that integrate diverse microbe, drug, and metabolite nodes. Specifically, based on the different types of current node, this model developed two different walk strategies, including metabolite as the current node type and drug/microbe as the current node type.

The first strategy is performed only when the type of current node is a metabolite. Suppose that the current node is $v\in V^{m}$ and the previous node is u, then the probability of selecting the next node n can be calculated as below:


$$p(n|v,u,M)=\frac{w_{vn}\cdot M_{Q(u,v)\;Q(v,n)}\cdot \alpha_{pq}(u,n)}{\sum_{k\in N(v)}M_{Q(u,v)\;Q(v,k)}\cdot \alpha_{pq}(u,k)}$$

where N(v) is the neighboring node set of the node v; Q(u,v) is the edge-type between u and v; M is an edge-type transition matrix of the heterogeneous graph G we construced, $M_{i,j}$ refers to the transition weight between edge-types i and j. The generation of M is based on the correlations of edge-types within the network and is obtained through an Expectation-Maximization (EM) framework aimed at optimizing the edge-type transition matrix (Gao *et al.* 2019, Bang *et al.* 2023). $w_{vn}$ denotes the weight of the edge(u,n), which is set to 1 by default. $\alpha_{pq}(u,k)$ is a normalization factor used to control the transition probabilities, which is set to 1.

The second strategy is performed when the type of current node is drug/microbe. The basic idea of this strategy is that we expect that if the current node has edges with metabolites and has no neighboring same-type nodes with high similarity, then the current node has a high probability of transferring to its neighboring metabolites. The biological insight of this strategy is that, as metabolites can act as mediators in complex drug-microbe interactions, the microbe–metabolite–drug walk can provide more mechanistic information about microbe–drug associations. To achieve this strategy, we skillfully design the probability function ρ(v), the details are as follows.

Suppose that the current node is v with a node type P(v)∈{drug, microbe} and the previous node is u, there is (i) a probability of p(n|v,S,ρ) to select the next node n with P(n)=P(v) or (ii) a probability of p(n|v,u,ρ) to select the next node n with node type P(n)=metabolite. For the scenario (i), the probability is calculated as $p(n|v,S,\rho )=\rho (v)\cdot \frac{S_{v,n}}{\sum_{k\in N_{s}(v)}S_{v,k}}$, where $N_{s}(v)$ is the neighboring node set of node v that share the same node type as v; $S_{v,n}$ is the similarity between nodes v and n. For the scenario (ii), the probability is calculated as $p(n|v,u,\rho )=(1-\rho (v))\cdot \frac{w_{vn}\cdot \alpha_{pq}(u,n)}{\sum_{k\in N(v)}\alpha_{pq}(u,k)}$, where N(v) is the neighboring metabolite node set of node v; $w_{vn}$ denotes the weight of the edge (v,n), which is set to 1 by default. $\alpha_{pq}(u,k)$ is a normalization factor used to control the transition probabilities, which is set to 1. Specifically, ρ(v) is defined as:


ρ(v)={1, T=0f(Svmax), T≠0

where $T=\;\sum_{j}{SM}_{v,j}$ when P(v)=microbe or $T=\;\sum_{j}{DM}_{v,j}$ when P(v)=drug; T=0 indicates that node v has no edges with any metabolites; we use the function $f(x)=\frac{1}{1+e^{-k(x-t)}}$ with k=100 and t=0.85, where f(x) approaches 1 when x>t, and approaches 0 when x<t; $S_{v_{\max}}$ is the highest similarity of the neighboring nodes of v.

### 2.4 Embedding generation

The Skip-gram model is widely used for learning continuous feature representations of nodes in the random walk-generated node sequences (Mikolov *et al.* 2013). However, the Skip-gram model does not consider the different types of nodes during the training steps, e.g. negative sampling step, thereby generating embedding vectors that are not aware of the node types. Here, for the heterogeneous node sequences acquired from the random walk model, we adopted a node-type-aware heterogeneous Skip-gram approach, which is an improvement of the Skip-gram model, to generate node embedding vectors (Bang *et al.* 2023). The embedding dimension and the window size of the heterogeneous Skip-gram approach we used are 1024 and 9.

### 2.5 MDA prediction

After obtaining the embedding vectors of all microbe and drug nodes in the MMD heterogeneous graph, we computed the embedding for each microbe–drug node pair. Specifically, we adopted element-wise multiplication of the two embeddings within each pair, as this operation is more suitable for capturing the metabolic commonalities between microbes and drugs than element-wise subtraction (Fig. S1, available as supplementary data at *Bioinformatics* online). The embedding $d_{uv}$ of the node pair of the microbe u and drug v is calculated as follows:


$$d_{uv}=z_{u}^{T}\cdot z_{v}$$

where $z_{u}$,$\;z_{v}\in R^{n}$ refers to the embedding vectors of microbe u and drug v.

We used the XGBoost classifier to predict microbe–drug associations by inputting the calculated embeddings of each microbe–drug node pair. XGBoost is a widely used machine learning algorithm across various application domains, including computational drug discovery (Chen and Guestrin 2016). As an ensemble model, XGBoost integrates multiple weak learners—specifically, decision trees—using a boosting algorithm to form a stronger and more accurate model. The boosting process involves training each weak learner sequentially, focusing on the residual errors of its predecessors, thus progressively improving the model’s overall performance. Specifically, we evaluated the performance of different classifiers in the results section, which also demonstrates the best performance of XGBoost. The loss function of XGBoost is given as follows:


$$\begin{array}{c} L\mathrm{oss}=\sum_{i=1}^{N}l(y_{i},\hat{y_{i}})+\sum_{k=1}^{K}\Omega (f_{k})\\ \begin{array}{c} l(y_{i},\hat{y_{i}})=-[y_{i}\log\left(\hat{y_{i}}\right)+(1-y_{i})\log(1-\hat{y_{i}})]\\ \Omega (f)=\gamma T+\frac{1}{2}\lambda {\left||w|\right|}^{2} \end{array} \end{array}$$

where $y_{i}$ represent the true label value for the ith microbe–drug pair, if $y_{i}=1$ refers to that this microbe–drug pair is a real microbe–drug association; $\hat{y_{i}}$ represent the predicted label value for the ith microbe–drug pair; N is the number of microbe–drug pairs; K=564 is the number of weak classifiers; $f_{k}$ is the kth weak classifier (CART decision tree); w is the vector of leaf scores in tree T; γ and λ take default parameters.

### 2.6 Experimental setup

We performed two cross-validations (CVs), i.e. 5-fold and 10-fold, across three different datasets (i.e. MDAD, aBiofilm, and MASI) to evaluate the performance of MetaMDA. Considering the 5-fold CV, we equally divided all the observed MDAs into five groups, and iteratively used one group for testing and the remaining four groups for training. We used four performance metrics, including Area Under the Receiver Operating Characteristic Curve (AUROC), Area Under the Precision-Recall Curve (AUPR), Accuracy (ACC), and F1-score during cross-validation, which are widely used for pairwise association predictions. Specifically, AUROC evaluates a model’s ability to distinguish between positive and negative classes across different thresholds. AUPR assesses model performance in imbalanced datasets by focusing on precision and recall trade-offs. Additionally, the calculation of ACC and F1-score is shown as follows:


$$\begin{array}{c} A\mathrm{CC}=\frac{\mathrm{TP}+\mathrm{TN}}{\mathrm{TP}+\mathrm{TN}+\mathrm{FP}+\mathrm{FN}}\\ \begin{array}{c} F1-\mathrm{score}=2\times \frac{\mathrm{Precision}\times \mathrm{Recall}}{\mathrm{Precision}+\mathrm{Recall}}\\ \begin{array}{c} \mathrm{Precision}=\frac{\mathrm{TP}}{\mathrm{TP}+\mathrm{FP}}\\ \mathrm{Recall}=\frac{\mathrm{TP}}{\mathrm{TP}+\mathrm{FN}} \end{array} \end{array} \end{array}$$

where TP means the number of predicted MDAs which are verified by the known database; FP means the number of predicted MDAs which are not verified by the known database; TN means the number of MDAs that are neither predicted nor verified by the known database; FN means the number of MDAs verified by the known database but not predicted.

## 3 Results

### 3.1 MetaMDA demonstrates superior performance in MDA prediction

**Figure 2.**
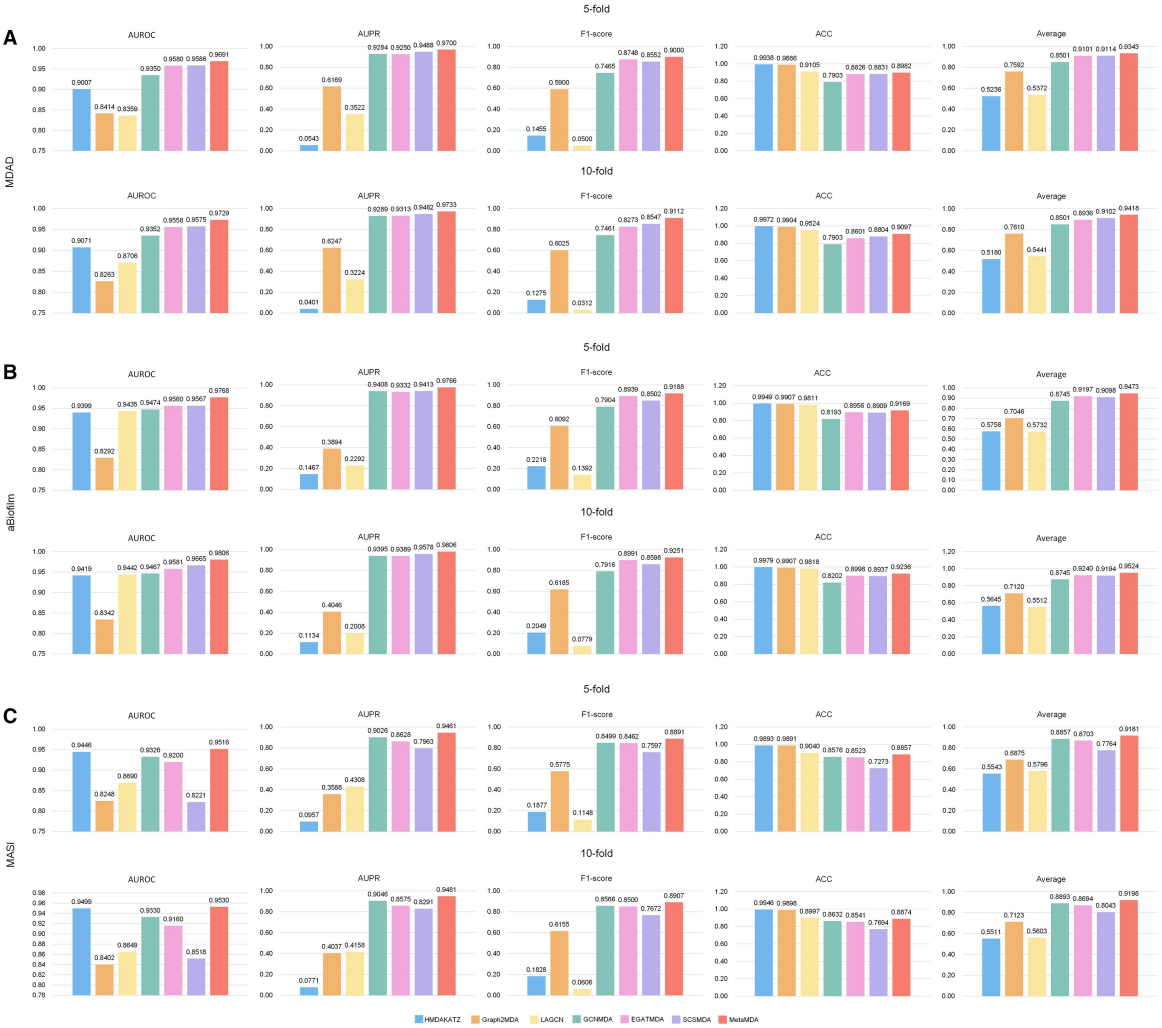
Performance comparison between MetaMDA and existing methods on the (A) MDAD, (B) aBiofilm, and (C) MASI datasets.

To evaluate the performance of MetaMDA, we compared it with six state-of-the-art methods using their original default parameters. All methods were tested on three benchmark datasets—MDAD, aBiofilm, and MASI—to assess effectiveness and robustness. As a result, MetaMDA consistently achieved the best performance across various evaluation metrics and datasets (Fig. 2 and Tables S2–S4, available as supplementary data at *Bioinformatics* online).

For the MDAD database, MetaMDA achieves the best prediction performance. Figure 2A shows that our method achieves an average score of 0.9343 across the four metrics, outperforming the second-best method, SCSMDA, by 2.51% and the third-best method, EGATMDA, by 2.66% based on the results of 5-fold CV. Similarly, based on the results of 10-fold CV, our method achieves an average score of 0.9418 across the four metrics, which is 3.47% higher than that of the second-best method, SCSMDA, and 5.39% higher than that of the third-best method, EGATMDA. Specifically, our method achieves the highest average AUROC, AUPR, and F1-score metric. For the metric ACC, the scores of methods HMDAKATZ, Graph2MDA, and LAGCN are higher than our method. It is noteworthy that although these three methods achieve high ACC, their AUROC, AUPR, and F1-score are relatively lower. This is because they treat all unlabeled MDAs as negative samples, leading to class imbalance. Moreover, on the aBiofilm and MASI datasets, MetaMDA also achieves the best predictive performance under both 5-fold and 10-fold cross-validation (Fig. 2B and C). All these results confirm that our method outperformed all six state-of-the-art methods consistently on different datasets, indicating MetaMDA is an effective and robust computational model for predicting MDAs.

### 3.2 Ablation study of MetaMDA

**Figure 3.**
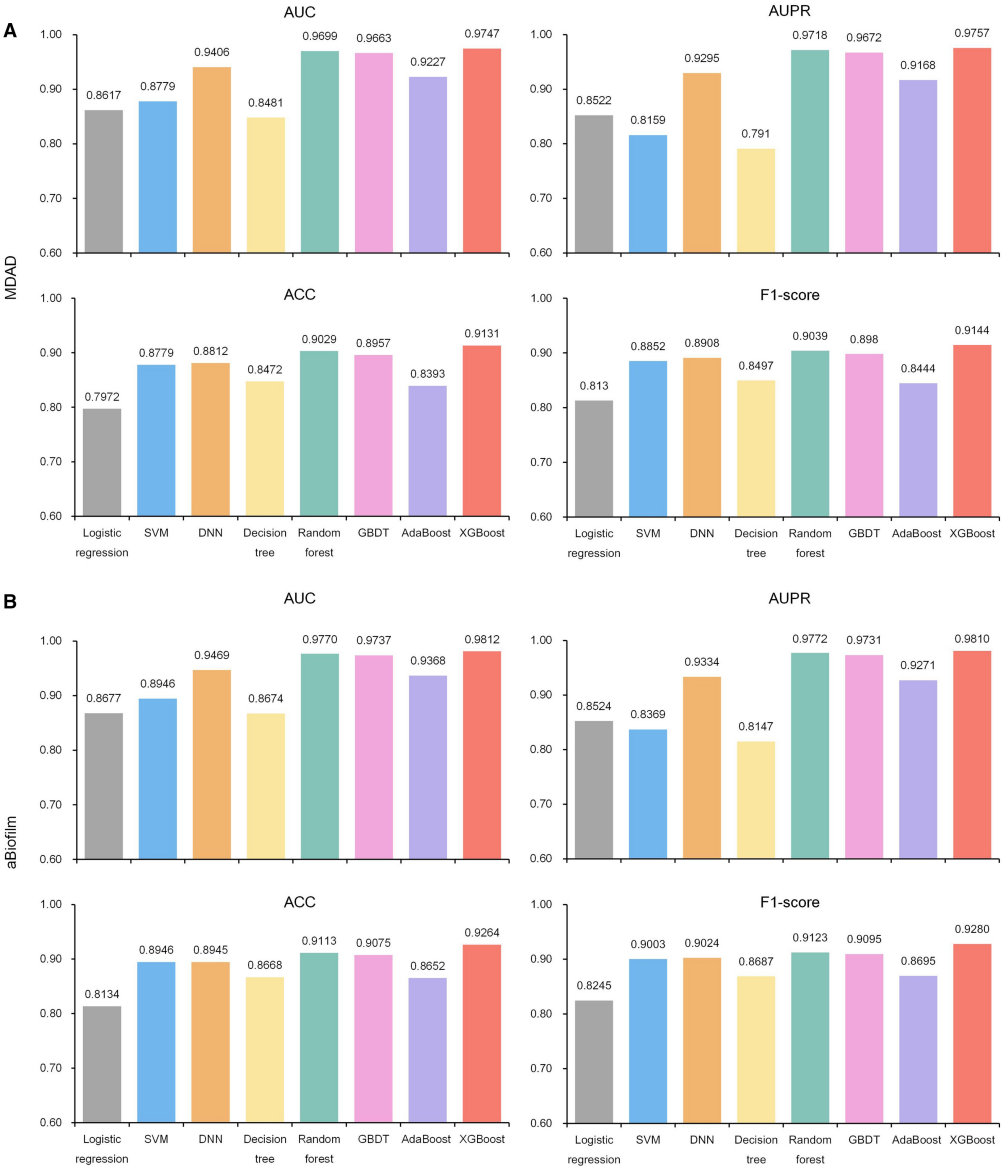
Comparative analysis across eight different classifiers on the (A) MDAD and (B) aBiofilm datasets.

We conducted two ablation studies to evaluate the contributions of key components in our framework: classifier ablation and similarity ablation. In the classifier ablation study, we assessed the impact of different classification models on overall performance. After acquiring the embedding representations for all nodes in the MMD heterogeneous graph by the heterogeneous Skip-gram approach, a specific classifier is applied to predict MDAs. To choose the best classifier, we test eight different classifiers for predicting MDAs. The performance of these eight classifiers across four metrics on the MDAD and aBiofilm dataset, based on 10-fold CV, is shown in Fig. 3. For the MDAD dataset, XGBoost achieves the highest performance among all classifiers across each performance metric, with an average score of 0.9445, followed by random forest with an average score of 0.9371 (Fig. 3A). In contrast, logistic regression exhibits the lowest performance, with an average score of 0.8310. For the aBiofilm dataset, XGBoost consistently outperformed all other classifiers across evaluation metrics, achieving an average score of 0.9542, with random forest ranking second at 0.9445 (Fig. 3B). This result demonstrated that XGBoost has exceptional effectiveness in binary classification tasks involving multiple features. Specifically, the best performance of XGBoost can be attributed to its efficient gradient boosting framework and built-in regularization, which collectively enhance generalization and reduce overfitting (Chen and Guestrin 2016). In the similarity ablation study, we examined how different similarity measures used in constructing the MMD heterogeneous graph affect the performance of MDA prediction (Supplementary Method S3, available as supplementary data at *Bioinformatics* online). The best performance is achieved when both Gaussian interaction profile kernel-based similarity and functional similarity are used (Table S5, available as supplementary data at *Bioinformatics* online), indicating that incorporating prior knowledge from data mining and biological associations enhances the model’s ability to capture complex relationships and improve prediction accuracy.

### 3.3 Parameter sensitivity analysis of MetaMDA

**Figure 4.**
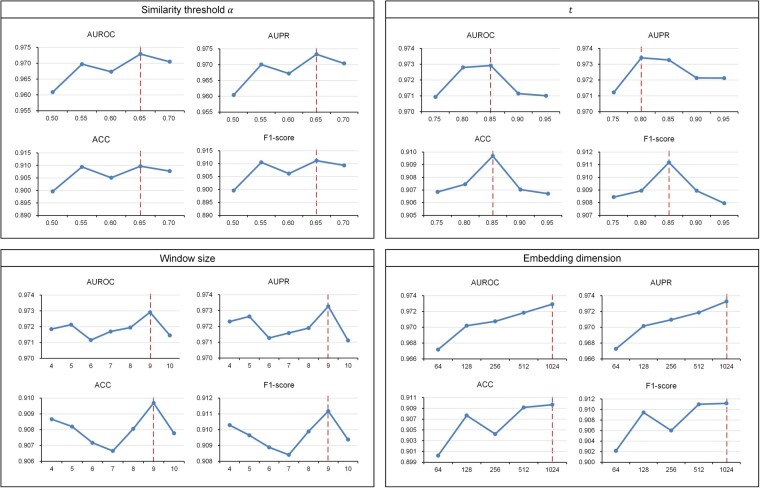
Parameter sensitivity of MetaMDA w.r.t. similarity threshold α, parameter t, window size, and embedding dimension. The dashed line represents the highest value.

Several key parameters influence the performance of MetaMDA. Here, we investigated four key parameters: the similarity threshold α in constructing the MMD heterogeneous graph, the parameter t of the random walk model, as well as the embedding dimension and the window size of the heterogeneous Skip-gram approach. We choose the values from {0.50,0.55,0.60,0.65,0.70} for the similarity threshold α, the values from {0.75,0.80,0.85,0.90,0.95} for the parameter t, the values from {4,5,6,7,8,9,10} for window size, and the values from {64,128,256,512,1024} for embedding dimension. The experiments are conducted using the MDAD dataset, with performance evaluated based on AUROC, AUPR, ACC, and F1-score. It should be noted that we perform the parameter sensitivity analysis using a 10-fold CV for all parameters. Figure 4 shows the results for the parameter sensitivity analysis. MetaMDA achieves the best performance when α=0.65, t=0.85, the window size is 9, and the embedding dimension is 1024. While t=0.85 does not yield the highest AUPR value, it provides the best overall performance across other evaluation metrics. For the embedding dimension, we selected 1024 instead of a higher value to prevent overfitting and avoid excessive computational costs.

### 3.4 MetaMDA accurately identifies MDAs for tetracycline and vancomycin

To further validate the prediction performance of MetaMDA, we conducted case studies on two widely used antimicrobial drugs, tetracycline and vancomycin, using the MDAD database. For each of them, all known entries are reset to unknown, and all candidate microbes are ranked in descending order based on their predicted scores. To evaluate our model’s performance, we examine whether the top 10 and top 20 ranked candidate microbes have been previously validated by existing studies.

Drug tetracycline is a broad-spectrum antimicrobial agent effective against a wide range of microorganisms, including Gram-positive and Gram-negative bacteria. It is widely used to treat various bacterial infections, such as respiratory tract infections, acne, and certain zoonotic diseases (Chopra and Roberts 2001). An increasing number of studies have indicated that it has a close interaction with a wide range of human microbes. Common interactions include its antimicrobial activity, toxicity against microorganisms, and the resistance developed by microorganisms in response to it. For example, Doherty *et al.* reported that *Streptococcus pneumoniae* exhibits tetracycline resistance, primarily through a mechanism that protects the bacterial 30S ribosomal subunit from antibiotic binding (Doherty *et al.* 2000). Karami *et al.* found that resistance to tetracycline remains common in *E. coli* (Karami *et al.* 2006). As a result, among the top 10 and 20 predicted tetracycline-associated microbes, all 10 and 20 microbe–drug associations, respectively, have been validated by previously published literature. These remarkably high confirmation rates (100% for all) highlight the robustness of MetaMDA and its potential for real-world applications. Table 1 presents the top 20 predicted candidate microbes associated with tetracycline.

**Table 1.** The top 20 predicted tetracycline-associated microbes.

Microbes (top 1–10)	Evidence	Microbes (top 11–20)	Evidence
*Staphylococcus aureus*	PMID: 10837427	*Haemophilus influenzae*	PMID: 6604106
*Escherichia coli*	PMID: 16377681	*Streptococcus pneumoniae*	PMID: 11036009
*Pseudomonas aeruginosa*	PMID: 24409175	*Proteus vulgaris*	PMID: 36523753
*Candida albicans*	PMID: 18310042	*Staphylococcus epidermis*	PMID: 36350834
*Clostridium perfringens*	PMID: 20600224	*Streptococcus sanguis*	PMID: 2981772
*Propionibacterium acnes*	PMID: 28218057	*Enterococcus faecium*	PMID: 1854161
*Acinetobacter baumannii*	PMID: 25801348	*Bacteroides fragilis*	PMID: 15825387
*Bacillus subtilis*	PMID: 8962071	*Streptococcus pyogenes*	PMID: 10834960
*Listeria monocytogenes*	PMID: 37888979	*Vibrio cholerae*	PMID: 34362438
*Staphylococcus epidermidis*	PMID: 36350834	*Human immunodeficiency virus*	PMID: 9415714

Drug vancomycin is a potent glycopeptide antibiotic commonly prescribed for the treatment of serious bacterial infections (Mühlberg *et al.* 2020). Emerging evidence highlights that it interacts closely with a variety of human microorganisms. For instance, Cong *et al.* reported that vancomycin remains a first-line treatment for infections caused by methicillin-resistant *Staphylococcus aureus* (MRSA) (Cong *et al.* 2020). Best *et al.* demonstrated that vancomycin inhibits both growth and mucopeptide synthesis in *Bacillus subtilis* (Best and Durham 1964). As a result, 10 out of the top 10 and 19 out of the top 20 predicted tetracycline-associated microbes have been validated by previously published studies. These remarkably high confirmation rates (100% for the top 10 and 95% for the top 20) highlight the robustness of MetaMDA and underscore its strong potential for real-world applications. Table 2 presents the top 20 predicted candidate microbes associated with vancomycin.

**Table 2.** The top 20 predicted vancomycin-associated microbes.

Microbes (top 1–10)	Evidence	Microbes (top 11–20)	Evidence
*Escherichia coli*	PMID: 26853624	*Staphylococcus epidermidis*	PMID: 37537250
*Staphylococcus aureus*	PMID: 26853624	*Eggerthella lenta*	PMID: 25520446
*Listeria monocytogenes*	PMID: 34680788	*Amycolatopsis orientalis*	PMID: 24884615
*Bacillus subtilis*	PMID: 14165485	*Staphylococcus epidermis*	PMID: 37537250
*Pseudomonas aeruginosa*	PMID: 26980934	*Staphylococcus cohnii*	PMID: 35778767
*Streptococcus pneumoniae*	PMID: 10608791	*Enterococcus faecium*	PMID: 11294702
*Mycobacterium tuberculosis*	PMID: 33508482	*Staphylococcus caprae*	PMID: 24975594
*Candida* spp.	PMID: 25631674	*Staphylococcus chromogenes*	PMID: 35778767
*Enterococcus faecalis*	PMID: 11294702	*Hepatitis C Virus*	Unconfirmed
*Streptococcus mutans*	PMID: 29070798	*Staphylococcus capitis*	PMID: 12089273

### 3.5 MetaMDA’s unique ability to predict MDAs involving unlabeled drugs

Furthermore, we demonstrated the unique capability of MetaMDA to predict MDAs involving microbes or drugs absent from the labeled MDA dataset, which is an ability lacking in existing methods. As a case study, we investigated the non-antibiotic drug acarbose, which has been shown to exert significant effects on the gut microbiome in both murine models and humans (Mohammadian *et al.* 2024, Zhang *et al.* 2024).

**Figure 5.**
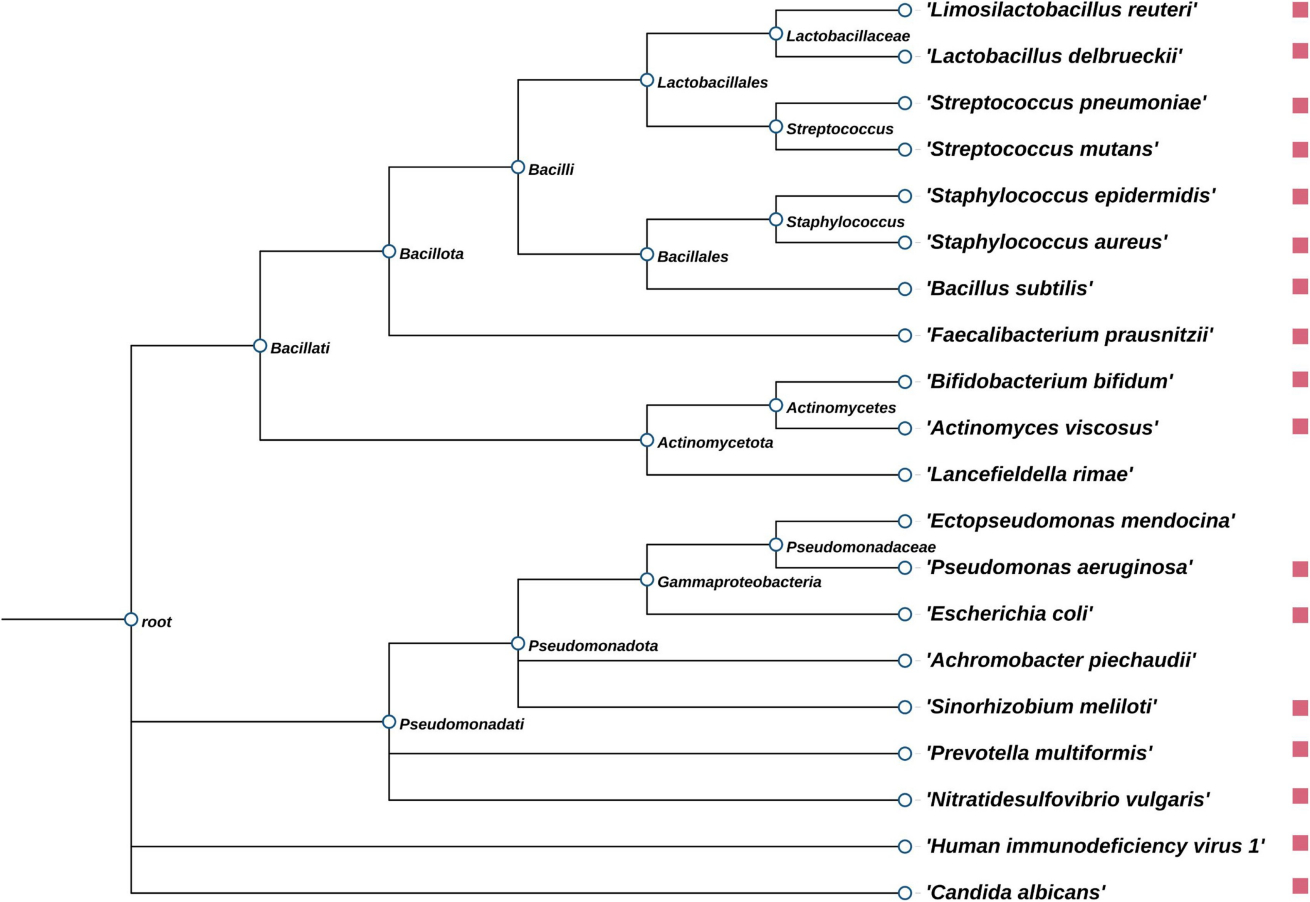
The phylogenetic relationship of the top 20 predicted acarbose-associated microbes. Microbes highlighted with rectangles have been confirmed in the literature to be associated with acarbose.

Acarbose, an antidiabetic drug not included in the labeled MDAs, is commonly used to treat type 2 diabetes mellitus (T2DM) (Zhang *et al.* 2022). Studies have shown that human gut microbes can inactivate acarbose through microbial-mediated degradation (Tian *et al.* 2023a). Additionally, the human microbiome harbors resistance mechanisms against acarbose (Balaich *et al.* 2021). These findings highlight the complex interactions between acarbose and gut microbes. Specifically, Liu *et al.* reported that acarbose can reduce *Pseudomonas aeruginosa* respiratory tract infection in type 2 diabetic mice (Liu *et al.* 2023). David *et al.* demonstrated that acarbose inhibits morphogenesis, adhesion, and invasion in *Candida albicans*, potentially through suppression of multiple related genes (David *et al.* 2024). Kumar *et al.* found that *Staphylococcus aureus* and *Staphylococcus epidermidis* may interact with acarbose via homologues of peptidyl-prolyl isomerase (PpiB), thereby influencing their biofilm formation ability (Kumar *et al.* 2019). Additionally, Whang *et al.* observed that acarbose administration increased the abundance of short-chain fatty acid (SCFA)-producing taxa such as *Faecalibacterium*, *Prevotella*, and *Lactobacillus* (Whang *et al.* 2019). As a result, 9 out of the top 10 and 17 out of the top 20 predicted acarbose-associated microbes have been validated by previously published studies. These notably high confirmation rates (90% for the top 10 and 85% for the top 20) demonstrate the unique capability of MetaMDA to predict MDAs involving microbes or drugs absent from the labeled MDA dataset, thereby enabling the discovery of more comprehensive associations. Figure 5 and Table S6, available as supplementary data at *Bioinformatics* online present the top 20 predicted candidate microbes associated with acarbose. Specifically, the phylogenetic relationship of the top 20 predicted acarbose-associated microbes was visualized using iTOL (Letunic and Bork 2024).

### 3.6 MetaMDA provides biological mechanistic insights for MDA prediction

**Figure 6.**
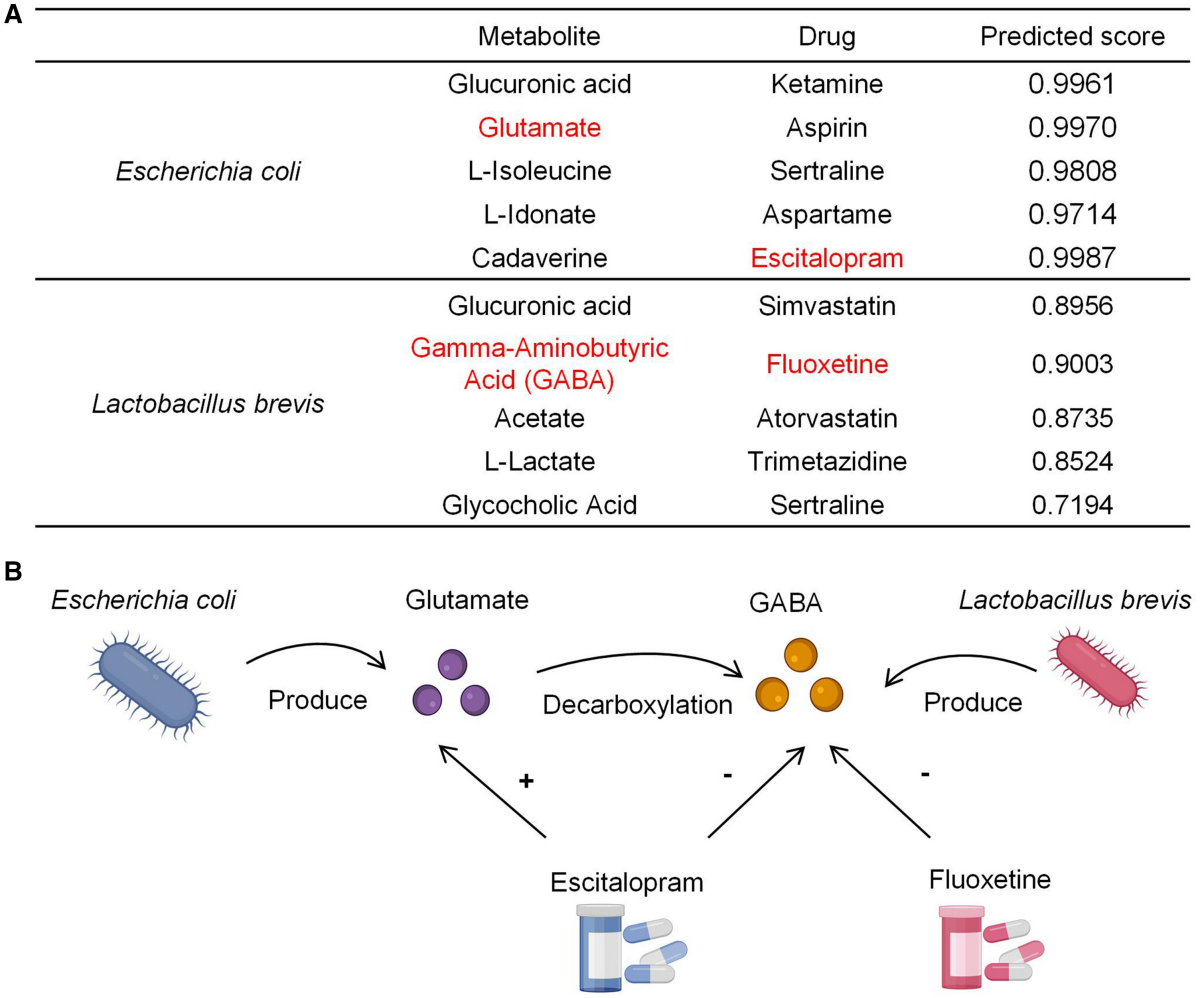
Mechanism analysis of MetaMDA. (A) The most frequently occurring drugs and metabolites in node sequences involving *E. coli* or *Lactobacillus brevis* were generated by the random walk model. The predicted scores between the drugs and their corresponding microbes are also presented here. (B) The mechanism of interaction between *E. coli* and escitalopram, and between *Lactobacillus brevis* and fluoxetine.

To explore the biological interaction mechanisms between microbes and drugs, we constructed an MMD heterogeneous graph by integrating a shared metabolite space and performed random walks on it. Here, we investigated the resulting node sequences from the random walk model to demonstrate the effectiveness of our method in uncovering the underlying interactions between microbes and drugs. Specifically, we separately searched for node sequences containing only *E. coli* or *Lactobacillus brevis*, two common human-associated microbes, and identified the most frequently occurring drugs and metabolites in these sequences. The results showed that the drugs most frequently appearing in the node sequences for *E*. *coli* and *L*. *brevis* exhibited high predicted interaction scores (over 0.7) with their corresponding microbes, demonstrating that the generated node sequences effectively capture microbe–drug interaction information (Fig. 6A). Notably, for *E*. *coli*, glutamate and escitalopram were among the top five associated metabolites and drugs, respectively. Several studies have suggested that glutamate produced by *E*. *coli* and escitalopram may have synergistic effects in the development and treatment of neurological and psychiatric diseases. Specifically, certain strains of *E*. *coli* have been reported to produce glutamate, which serves as a major excitatory neurotransmitter in the central nervous system and can be converted into Gamma-Aminobutyric Acid (GABA), the major inhibitory neurotransmitter (Kern *et al.* 2025). Dysregulation of these neurotransmitters is linked to neurological and psychiatric disorders such as major depressive disorder (MDD) (Sarawagi *et al.* 2021). Escitalopram, an antidepressant, may enhance glutamate transmission by reducing GABA-mediated inhibition (Pehrson and Sanchez 2014, Zsido *et al.* 2022). These findings highlight the complex relationship between *E*. *coli* and escitalopram in the development and treatment of neurological and psychiatric diseases (Fig. 6B). Additionally, for *L*. *brevis*, GABA and fluoxetine were among the top five associated metabolites and drugs, respectively. Research has shown that *L*. *brevis* from the human intestine produces GABA, while fluoxetine—a widely used selective serotonin reuptake inhibitor (SSRI) for psychiatric disorders—can reduce GABAergic inhibition in the adult brain (Begenisic *et al.* 2014, Kern *et al.* 2025) (Fig. 6B). All these results demonstrated that, unlike current methods, which primarily address MDA prediction as a computational link prediction problem, our approach provides deeper insights into the underlying biological interaction mechanisms between microbes and drugs.

## 4 Discussion

Recent studies have indicated that human-associated microorganisms play key roles in physiological processes and are linked to a wide range of diseases (Sepich-Poore *et al.* 2021, El Tekle and Garrett 2023). More importantly, microorganisms in the gastrointestinal tract and other niches can also contribute to cancer development and progression by interacting with the host immune system (Cullin *et al.* 2021). Predicting MDAs can advance drug development and promote personalized medicine (Zimmermann *et al.* 2019, Zhao *et al.* 2023). In this study, we propose an explainable computational framework, MetaMDA, for predicting MDAs. MetaMDA first constructs a heterogeneous graph that integrates microbes, metabolites, and drugs by introducing a metabolite space shared by both microbes and drugs, thereby facilitating the discovery of underlying biological interaction mechanisms. To effectively capture multi-type node features on a unified scale, we develop a random walk algorithm with tailored transition probabilities across different node types. Finally, this integrated framework enables more comprehensive and interpretable prediction of MDAs. The results demonstrate that our method consistently outperforms all state-of-the-art approaches across multiple datasets, achieving an average relative improvement of 26% (Table S7, available as supplementary data at *Bioinformatics* online). Ablation studies and parameter sensitivity analysis were conducted to validate the effectiveness of each key component within the MetaMDA framework. These findings indicate that MetaMDA is a robust and effective computational model for predicting MDAs. More importantly, we showcased MetaMDA’s unique ability to predict MDAs involving microbes or drugs that are absent from the labeled MDAs by investigating associations related to acarbose. Furthermore, analysis of the node sequences generated by the random walk model associated with *E*. *coli* revealed that the synergistic effect of *E*. *coli*-derived glutamate and escitalopram influences neurotransmitter balance, thereby impacting the treatment of psychiatric disorders. This finding demonstrates that MetaMDA provides deeper insights into the underlying biological interaction mechanisms between microbes and drugs.

In the future, there remains considerable potential to enhance our prediction model. First, we plan to construct a more comprehensive microbe–metabolite–drug (MMD) heterogeneous graph by incorporating additional microbe–metabolite and drug–metabolite interactions from newly published literature. For instance, GutCP, an ecology-based computational method (Goyal *et al.* 2021), can be used to predict previously untested cross-feeding interactions in the human gut microbiome, thereby expanding the microbe–metabolite edge set. Second, additional biological similarities can be integrated into the graph construction process. For example, the genomic content network (GCN)—a bipartite graph connecting microbes to the genes within their genomes—can be utilized to compute microbial genetic similarities based on GCN’s topological properties (Tian *et al.* 2020). Third, compared with randomly selecting negative samples from unlabeled MDAs, developing a more reliable negative sample selection strategy can further enhance MDA prediction performance. Recently, Ma *et al.* proposed a “guilty-by-association”-based negative sampling approach (Ma *et al.* 2023), and our computational experiments demonstrated that this method can improve the performance of MDA prediction (Table S8, available as supplementary data at *Bioinformatics* online). Finally, beyond MDA prediction, the learned embeddings of microbes and drugs, which incorporate information from drugs, metabolites, and microbial interactions, hold promise for broader biological and pharmacological applications.

## Supplementary Material

btaf649_Supplementary_Data

## Data Availability

The data and source code for MetaMDA are available on Zenodo https://doi.org/10.5281/zenodo.17348446 and GitHub https://github.com/wqlyt17/MetaMDA.
