# Supplementary material for: MetaMDA: explainable prediction of microbe–drug association utilizing random walks on a microbe–metabolite–drug heterogeneous network
Source: Bioinformatics. 2025 Dec 1;41(12):btaf649. doi: 10.1093/bioinformatics/btaf649 (PMC12714396; doi:10.1093/bioinformatics/btaf649)
Supplement: btaf649_Supplementary_Data [file btaf649_supplementary_data.docx]

**MetaMDA: explainable prediction of microbe-drug association utilizing random walks on a microbe-metabolite-drug heterogeneous network**

Qi Wang^1†^, Shuting Chen^1†^, Xintian Miao^1^, Yuntao Liu^1^, Bingqiang Liu^1,2,3,*^

^1^School of Mathematics, Shandong University, Jinan, Shandong 250100, China

^2^Shandong National Center for Applied Mathematics, Jinan, Shandong 250100, China

^3^State Key Laboratory of Cryptography and Digital Economy Security, Shandong University, Jinan, Shandong 250100, China

^*^ Correspondence: bingqiang@sdu.edu.cn (B. Liu)

†Equal contribution.

**Supplementary Methods**

**1. The calculation of the Gaussian interaction profile kernel-based similarity for the microbe-metabolite matrix** $\boldsymbol{SM}$

Here, we denote the $i$-th and $j$-th row in microbe-metabolite matrix $SM$ as $SM(v_{i}^{s})$ and $SM(v_{j}^{s})$. Thus, the Gaussian interaction profile kernel-based similarity for microbe $v_{i}^{s}$ and $v_{j}^{s}$ is calculated as:

$$GM\left( v_{i}^{s},v_{j}^{s} \right)=exp(-\eta_{s}{||SM\left( v_{i}^{s} \right)-SM(v_{j}^{s})||}^{2})$$

where, $\eta_{s}=\eta_{s}^{'}/(\frac{1}{N_{s}}\sum_{i=1}^{N_{s}} {||SM\left( v_{i}^{s} \right)||}^{2})$ is the normalized kernel bandwidth, $\eta_{s}^{'}$ is always set to 1; $N_{s}$ is the number of rows in matrix $SM$.

**2. The calculation of the Gaussian interaction profile kernel-based similarity for drug-metabolite matrix** $\boldsymbol{DM}$

Here, we denote the $i$-th and $j$-th row in drug-metabolite matrix $DM$ as $DM(v_{i}^{d})$ and $DM(v_{j}^{d})$. Thus, the Gaussian interaction profile kernel-based similarity for drug $v_{i}^{d}$ and $v_{j}^{d}$ is calculated as:

$$GM\left( v_{i}^{d},v_{j}^{d} \right)=exp(-\eta_{s}{||DM\left( v_{i}^{d} \right)-DM(v_{j}^{d})||}^{2})$$

where, $\eta_{s}=\eta_{s}^{'}/(\frac{1}{N_{d}}\sum_{i=1}^{N_{d}} {||DM\left( v_{i}^{d} \right)||}^{2})$ is the normalized kernel bandwidth, $\eta_{s}^{'}$ is always set to 1; $N_{d}$ is the number of rows in matrix $DM$.

**3. Comparison with different similarity measures**

An ablation study was conducted to evaluate the impact of each aspect of similarity for MDA prediction. MetaMDA-Gaussian uses Gaussian-interaction profile-kernel-based similarity. Specifically, when constructing an MMD heterogeneous graph, a microbe-microbe edge is created if the average Gaussian-interaction profile-kernel-based similarity for the microbe-drug matrix and the microbe-metabolite matrix between a pair of microbes is over a similarity threshold $\alpha$. If either the genomic or evolutionary similarity is zero, the non-zero value is used instead of the average. Similarly, a drug-drug edge is created if the average Gaussian-interaction profile-kernel-based similarity for the microbe-drug matrix and drug-metabolite matrix between a pair of drugs is over a similarity threshold $\alpha$. MetaMDA-Function uses functional similarity. For microbes, a microbe–microbe edge is added if the average of genomic and evolutionary similarities between two microbes exceeds $\alpha$. If either the genomic or evolutionary similarity is zero, the non-zero value is used instead of the average. For drugs, a drug–drug edge is added if the functional similarity between two drugs exceeds $\alpha$. MetaMDA integrates both Gaussian-interaction profile-kernel-based similarity and functional similarity. In this setting, a microbe–microbe or drug–drug edge is generated if the average of the two similarity measures exceeds the threshold $\alpha$.

**Supplementary Figures**

**
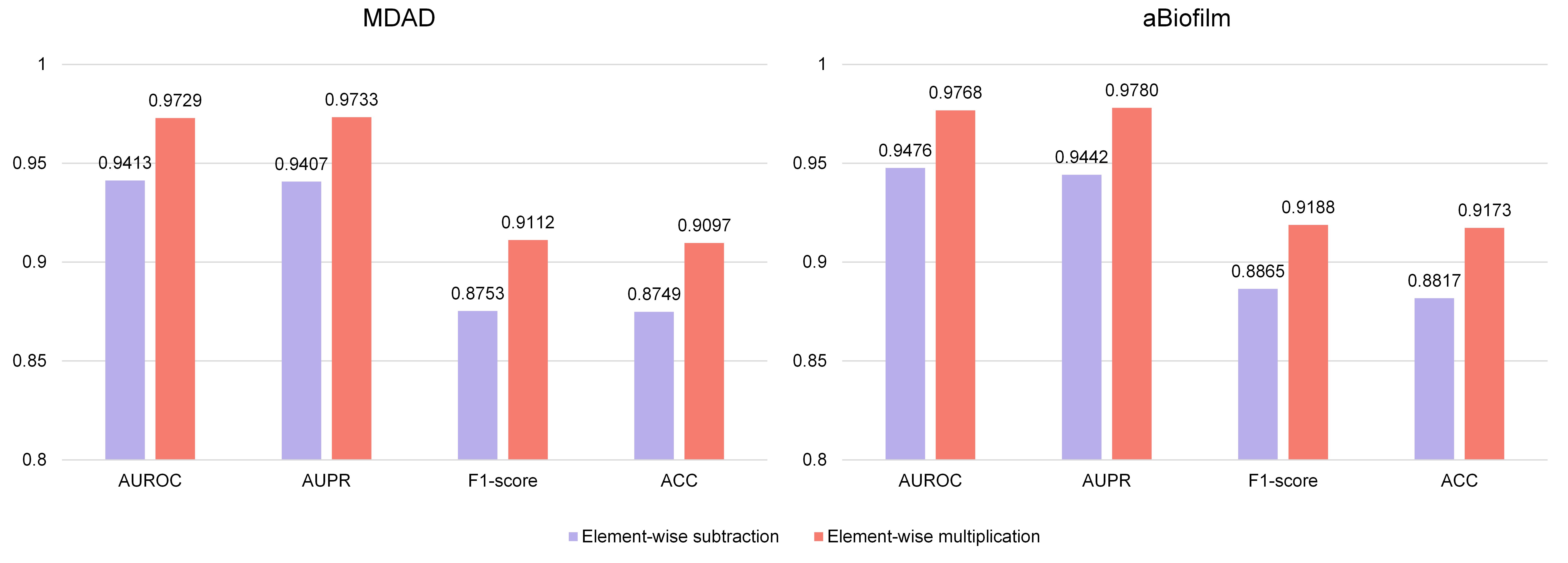
**

**Supplementary Figure S1.** Performance comparison of element-wise multiplication operation versus element-wise subtraction operation in computing the embedding of each microbe–drug node pair on the MDAD and aBiofilm datasets.

**Supplementary Tables**

**Supplementary Table S1.** The details of the constructed MMD heterogeneous graph for different datasets.

|  | Microbes | Metabolites | Drugs | Microbe-metabolite edge | Drug-metabolite edge | Metabolite-metabolite edge |
| --- | --- | --- | --- | --- | --- | --- |
| MDAD | 672 | 7,721 | 1,616 | 5,130 | 1,994 | 9,603 |
| aBiofilm | 635 | 7,721 | 1,518 | 5,130 | 1,994 | 9,603 |
| MASI | 1,032 | 7,721 | 1,142 | 5,130 | 1,994 | 9,603 |

**Supplementary Table S2.** The standard deviation values for each method across different performance metrics on MDAD.

|  |  | KATZMDA | Graph2MDA | LAGCN | GCNMDA | EGATMDA | SCSMDA | MetaMDA |
| --- | --- | --- | --- | --- | --- | --- | --- | --- |
| 5-fold | ACC | 0.0006 | 0.0025 | 0.0830 | 0.0017 | 0.0101 | 0.0053 | 0.0012 |
|  | AUROC | 0.0022 | 0.0157 | 0.0263 | 0.0018 | 0.0041 | 0.0015 | 0.0021 |
|  | AUPR | 0.0008 | 0.0062 | 0.0107 | 0.0021 | 0.0065 | 0.0022 | 0.0025 |
|  | F1-score | 0.0035 | 0.0179 | 0.0036 | 0.0016 | 0.0137 | 0.0066 | 0.0012 |
| 10-fold | ACC | 0.0002 | 0.0010 | 0.0057 | 0.0013 | 0.0258 | 0.0036 | 0.0020 |
|  | AUROC | 0.0013 | 0.0177 | 0.0227 | 0.0020 | 0.0048 | 0.0016 | 0.0011 |
|  | AUPR | 0.0012 | 0.0196 | 0.0191 | 0.0035 | 0.0108 | 0.0027 | 0.0010 |
|  | F1-score | 0.0022 | 0.0195 | 0.0040 | 0.0011 | 0.0603 | 0.0034 | 0.0018 |

**Supplementary Table S3.** The standard deviation values for each method across different performance metrics on aBiofilm.

|  |  | KATZMDA | Graph2MDA | LAGCN | GCNMDA | EGATMDA | SCSMDA | MetaMDA |
| --- | --- | --- | --- | --- | --- | --- | --- | --- |
| 5-fold | ACC | 0.0001 | 0.0018 | 0.0023 | 0.0012 | 0.0021 | 0.0048 | 0.0032 |
|  | AUROC | 0.0006 | 0.0096 | 0.0014 | 0.0013 | 0.0016 | 0.0011 | 0.0011 |
|  | AUPR | 0.0007 | 0.0431 | 0.0219 | 0.0017 | 0.0037 | 0.0026 | 0.0016 |
|  | F1-score | 0.0022 | 0.0389 | 0.0068 | 0.0011 | 0.0023 | 0.0046 | 0.0030 |
| 10-fold | ACC | 0.0000 | 0.0010 | 0.0033 | 0.0005 | 0.0007 | 0.0048 | 0.0020 |
|  | AUROC | 0.0004 | 0.0066 | 0.0012 | 0.0011 | 0.0010 | 0.0026 | 0.0004 |
|  | AUPR | 0.0002 | 0.0078 | 0.0214 | 0.0022 | 0.0011 | 0.0043 | 0.0004 |
|  | F1-score | 0.0004 | 0.0118 | 0.0061 | 0.0005 | 0.0008 | 0.0083 | 0.0021 |

**Supplementary Table S4.** The standard deviation values for each method across different performance metrics on MASI.

|  |  | KATZMDA | Graph2MDA | LAGCN | GCNMDA | EGATMDA | SCSMDA | MetaMDA |
| --- | --- | --- | --- | --- | --- | --- | --- | --- |
| 5-fold | ACC | 0.0003 | 0.0034 | 0.0146 | 0.0018 | 0.0022 | 0.0099 | 0.0026 |
|  | AUROC | 0.0001 | 0.0213 | 0.0237 | 0.0028 | 0.0010 | 0.0064 | 0.0022 |
|  | AUPR | 0.0008 | 0.0611 | 0.0195 | 0.0072 | 0.0025 | 0.0072 | 0.0031 |
|  | F1-score | 0.0012 | 0.0635 | 0.0487 | 0.0016 | 0.0028 | 0.0055 | 0.0025 |
| 10-fold | ACC | 0.0001 | 0.0016 | 0.0243 | 0.0013 | 0.0007 | 0.0045 | 0.0016 |
|  | AUROC | 0.0000 | 0.0069 | 0.0371 | 0.0021 | 0.0015 | 0.0026 | 0.0018 |
|  | AUPR | 0.0003 | 0.0321 | 0.0150 | 0.0038 | 0.0031 | 0.0036 | 0.0027 |
|  | F1-score | 0.0005 | 0.0305 | 0.0372 | 0.0012 | 0.0013 | 0.0106 | 0.0016 |

**Supplementary Table S5.** Performance comparison among different similarities under 10-fold CV. The method with the highest performance is highlighted in red.

|  |  | MetaMDA-Function | MetaMDA-Gaussian | MetaMDA |
| --- | --- | --- | --- | --- |
| MDAD | ACC | 0.7872 | 0.9094 | 0.9097 |
|  | AUROC | 0.8484 | 0.9721 | 0.9729 |
|  | AUPR | 0.8597 | 0.9720 | 0.9733 |
|  | F1-score | 0.7810 | 0.9112 | 0.9112 |
| aBiofilm | ACC | 0.7872 | 0.9224 | 0.9236 |
|  | AUROC | 0.8375 | 0.9790 | 0.9806 |
|  | AUPR | 0.8552 | 0.9785 | 0.9806 |
|  | F1-score | 0.7790 | 0.9241 | 0.9251 |

**Supplementary Table S6.** The top 20 predicted acarbose-associated microbes.

| Microbe | Evidence | Microbe | Evidence |
| --- | --- | --- | --- |
| *Pseudomonas aeruginosa* | PMID: 38098038 | *Bifidobacterium bifidum* | PMID: 35855337 |
| *Escherichia coli* | PMID: 10198028 | *Sinorhizobium meliloti* | PMID: 15939574 |
| *Human immunodeficiency virus 1* | PMID: 16075303 | *Ectopseudomonas mendocina* | Unconfirmed |
| *Staphylococcus aureus* | PMID: 30675370 | *Achromobacter piechaudii* | Unconfirmed |
| *Bacillus subtilis* | PMID: 1380303 | *Lactobacillus delbrueckii* | PMID: 30553752 |
| *Candida albicans* | PMID: 38789465 | *Lactobacillus reuteri* | PMID: 30553752 |
| *Streptococcus pneumoniae* | PMID: 32098834 | *Actinomyces viscosus* | PMID: 34819672 |
| *Staphylococcus epidermis* | PMID: 30675370 | *Prevotella multiformis* | PMID: 30553752 |
| *Lancefieldella rimae* | Unconfirmed | *Faecalibacterium prausnitzii* | PMID: 30553752 |
| *Streptococcus mutans* | PMID: 27105419 | *Desulfovibrio vulgaris* | PMID: 29962399 |

**Supplementary Table S7.** The relative improvement of MetaMDA over other methods across four performance metrics.

|  |  | HMDAKATZ | Graph2MDA | | LAGCN | GCNMDA | EGATMDA | SCSMDA | Average |
| --- | --- | --- | --- | --- | --- | --- | --- | --- | --- |
| MDAD | 5-fold | 0.7844 | | 0.2306 | 0.7392 | 0.0990 | 0.0266 | 0.0251 | 0.2241 |
|  | 10-fold | 0.8181 | | 0.2376 | 0.7309 | 0.1079 | 0.0539 | 0.0347 | 0.2330 |
| aBiofilm | 5-fold | 0.6452 | | 0.3445 | 0.6527 | 0.0832 | 0.0300 | 0.0412 | 0.2303 |
|  | 10-fold | 0.6872 | | 0.3376 | 0.7279 | 0.0891 | 0.0307 | 0.0359 | 0.2442 |
| MASI | 5-fold | 0.6562 | | 0.3353 | 0.5839 | 0.0366 | 0.0549 | 0.1826 | 0.3083 |
|  | 10-fold | 0.6690 | | 0.2913 | 0.6418 | 0.0343 | 0.0580 | 0.1436 | 0.3063 |

**Supplementary Table S8.** Performance comparison among different negative sample sampling methods under 10-fold CV.

|  |  | Random negative sampling | Guilty-by-association strategy |
| --- | --- | --- | --- |
| MDAD | ACC | 0.9131 | 0.9547 |
|  | AUROC | 0.9747 | 0.9891 |
|  | AUPR | 0.9757 | 0.9877 |
|  | F1-score | 0.9144 | 0.9506 |
| aBiofilm | ACC | 0.9264 | 0.9637 |
|  | AUROC | 0.9812 | 0.9921 |
|  | AUPR | 0.9810 | 0.9908 |
|  | F1-score | 0.9280 | 0.9606 |
